# A practical guide to simulation for an adaptive trial design with a single interim analysis

**DOI:** 10.1186/s13063-025-09100-5

**Published:** 2025-10-09

**Authors:** Kaushala S. Jayawardana, Michael Dymock, Robert K. Mahar, Julie A. Marsh, Katherine J. Lee

**Affiliations:** 1 Clinical Epidemiology and Biostatistics Unit, Murdoch Children’s Research Institute, Parkville, Victoria, Australia; 2 Department of Paediatrics, The University of Melbourne, Melbourne, Australia; 3 Wesfarmers Centre of Vaccines and Infectious Diseases, Telethon Kids Institute, Nedlands, WA Australia; 4 School of Population and Global Health, University of Western Australia, Perth, WA Australia; 5 Centre for Epidemiology and Biostatistics, Melbourne School of Population and Global Health, Faculty of Medicine, Dentistry, and Health Sciences, University of Melbourne, Melbourne, VIC Australia; 6 Methods and Implementation Support for Clinical and Health Research Hub, Faculty of Medicine, Dentistry, and Health Sciences, University of Melbourne, Melbourne, VIC Australia; 7 Centre for Child Health Research, The University of Western Australia, Perth, Australia

**Keywords:** Adaptive trials, Trial design, Simulation, Sample size

## Abstract

**Background:**

The demand for adaptive trial designs is growing because of their flexibility and the potential for efficiency gains over traditional fixed designs. Adaptive trials allow planned modifications to the design based on accumulating data. Simulation is imperative in designing adaptive trials because analytical power formulae cannot account for data-driven adaptations. Despite their popularity, the uptake of adaptive trials has been slowed by the lack of expertise and availability of training resources.

**Methods:**

In this tutorial, we demonstrate how to simulate data from a simple adaptive trial with a single interim analysis, summarise the simulations, and use these results to balance the type I error and power to inform the study design and to determine the expected sample size. The simulation code, based on a real trial in hyponatraemia in children, is provided in both R and Stata programming languages. The code is written in modules to improve comprehensibility and enable simple changes to generate a range of adaptive designs.

**Discussion:**

When using simulation to design an adaptive trial, the simulations must be tailored to the unique design requirements of the trial at hand. This tutorial provides a foundational framework designed to make the simulation process more accessible to both statisticians and clinicians.

**Supplementary Information:**

The online version contains supplementary material available at 10.1186/s13063-025-09100-5.

## Introduction

Adaptive trial designs are becoming increasingly important in medical research as they allow for prospectively planned modifications to one or more aspects of an ongoing clinical trial based on accumulating data, without sacrificing the trial validity and integrity [1–7]. The demand for these designs is growing because they are flexible and can provide efficiency gains over conventional designs, often in terms of cost or time [1, 3, 6, 8]. This flexibility is particularly beneficial in areas such as infectious diseases like COVID-19, oncology and rare diseases, where patient populations are small, and treatment effects need to be assessed rapidly [5, 9, 10]. The most common pre-planned modifications are changes to the sample size to declare treatment efficacy or futility (early stopping), ceasing randomisation to futile treatment arms (arm dropping), and modifying the allocation probabilities to each treatment arm (adaptive randomisation) [5, 6]. These modifications can improve resource efficiency if fewer participants receive inferior treatment/s, or if the trial requires fewer participants overall when compared with a traditional fixed design [6]. The group sequential design is an example of an adaptive design that incorporates multiple planned interim analyses, with pre-specified rules for early stopping [11]. A more complex example is the platform design, where multiple treatments are evaluated simultaneously, across a number of participant subgroups, under a single core protocol [12–15]. Platform designs may have the benefit of using the same control group across multiple research questions and the ability to add new interventions as funds or supplies become available, but require careful planning around adaptation criteria and analysis, and more complex statistical modelling to account for non-concurrent controls [16, 17].

Despite their advantages and popularity, the uptake of adaptive designs has been slow amongst clinical trialists. This may be due to the practical challenges in their design and implementation, poor access to design expertise, reservations about acceptance by regulatory authorities, stakeholders and funders, and the complexity of interpreting the results [2, 5, 6].

Adaptive designs can use either frequentist or Bayesian methods for design, inference, and decision making (or a combination thereof) [6, 11, 18]. In a frequentist design, the decision to stop the trial early for efficacy or futility is typically made by comparing the *p*-value for a treatment effect, calculated within a hypothesis testing framework, against pre-defined stopping boundaries [18]. For example, with a frequentist design the trial could be stopped for efficacy if the treatment effect *p*-value at an interim analysis is less than 0.005, a pre-defined threshold chosen to control the false positive rate (*α*, the probability of rejecting the null hypothesis when there is truly no difference between the treatment arms) [18–21]. In a Bayesian design, these decisions are typically guided by the posterior probability of clinically relevant treatment effects, e.g., for superiority this may be the probability that the relative risk is less than one [18]. For example, the trial could be stopped for efficacy if the posterior probability of treatment being superior to the control is greater than 0.95, where the pre-defined threshold is usually chosen to control the frequentist false positive rate [22]. Although type I error control is not formally required in a Bayesian design, it is common to report frequentist operating characteristics in these designs, particularly if the trial aims to satisfy regulatory requirements [6, 18, 23–27]. Therefore, most Bayesian adaptive designs are a hybrid of frequentist and Bayesian methods as they are designed based on frequentist operating characteristics such as power and type I error, but the interim analyses and adaptation criteria are based on Bayesian inference and decision rules [6, 28–31].

For simple adaptive designs, such as group sequential designs, established frequentist formulae can be used to determine the operating characteristics, such as power and type I error or the required sample size. However, many adaptive trials require computer simulation to estimate the operating characteristics and identify an efficient trial design. The operating characteristics will depend on the clinical phase of the trial and the degree of risk acceptable to the investigator, sponsor, and/or regulator, in addition to implementation feasibility [6, 32–35]. Simulation studies are widely used in statistics to evaluate and understand the performance of statistical methods [36, 37]. More recently, simulation has become pivotal in the design of innovative clinical trials [7, 38–40]. Simulation involves generating virtual (i.e., *computer generated and hypothetical*) trial data under different assumed clinical effects for the treatment and control arms, often referred to as scenarios [32]. Data for thousands of ‘virtual trials’ are generated and analysed and operating characteristics such as the power, type I error and sample size are summarised for each scenario. These scenarios can incorporate various design features such as the timing and number of interim analyses, the decision rules for trial adaptations, and the number of treatment arms. Setting these design features usually happens via an iterative process, where results from a growing number of scenarios are discussed amongst the statisticians and clinicians and the design features are updated for the next batch of simulations; a cycle that continues until acceptable operating characteristics are achieved. This iterative process facilitates the communication of important trial decisions, which in turn builds confidence in the design and analysis prior to recruiting the first participant [33, 35].

A range of software exists for conducting simulations for adaptive trials including stand-alone software (e.g., FACTS [41], ADDPLAN [42] and EAST [43]), packages within existing software such as R [44] (e.g., gsDesign [45], bayesCT [46], MAMS [47], asd [48], rpact [49]) and Stata [50] (e.g., nstage [51]), online trial simulators (e.g. HECT [52]) and custom written code that is sometimes available from the addendums to publications [34, 35, 53–55]. However, some software are limited in the availability of design options, while others may overwhelm the users with their availability of a wide range of design features [34]. Owing to the limited capabilities or flexibility and the complexity of the available software, experienced programmers often find it more efficient to write their own code [34]. This also offers the flexibility to deal with the unique nature of the wide variety of adaptive designs that may be used. However, there is currently a lack of guidance on developing code for conducting simulations, and on the general process for how these simulations are used to guide the trial design, although this approach may be unfamiliar to most clinicians and trial methodologists.

The aim of this tutorial is to provide a step-by-step guide on how to write code to simulate trial data and how to interpret the output for a range of scenarios to inform the design of a simple adaptive trial with a single interim analysis. This process will be useful to both statisticians and clinical trialists wishing to implement adaptive designs. We provide the simulation code in both R (within the main text) and Stata (in the supplementary material) using a modular coding structure to enhance comprehensibility and facilitate modifications to a range of adaptive designs. The provided code can serve as a foundation for generating simulations for any trial. However, it will need to be adapted to the specific design features of the trial—for example, by modifying parameters such as recruitment rate, allocation ratios, and stopping rule. We focus our attention on a frequentist example, but the code could be adapted to incorporate Bayesian decision making. We illustrate the simulation and design process using a real-world example of the Paediatric Intravenous Maintenance Solution in reducing the risk of hyponatraemia in children in hospital (PIMS) trial, published previously [56]. Although there were no adaptive elements in the original trial, we assume in this tutorial that the trial included a single interim analysis to illustrate the simulation and design processes.

We begin by providing details of the PIMS trial in section. “Illustrative example: the PIMS trial”. In section “The simulation process”, we outline the simulation process and explain the code required to generate the simulations. We use a modular structure and introduce subroutines or functions for generating the different aspects of the trial data, which we call ‘*building blocks*’. The building blocks are combined to produce a trial simulation that is run many times under a number of clinically relevant scenarios. In the section “Simulation outputs”, we discuss the outputs of the simulation and how these should be summarised and interpreted. We conclude with a discussion on balancing the design options against the investigator/sponsor/regulator risk strategies in the Section ‘Discussion’.

## Illustrative example: the PIMS trial

### Overview of the PIMS trial

The PIMS trial was a two-arm, parallel-group, randomised, double blind trial conducted at the Royal Children’s Hospital, Melbourne, Australia, to determine whether the use of a fluid solution with a higher sodium concentration reduced the risk of hyponatraemia compared with the use of a hypotonic solution. Participants were children aged 3 months to 18 years admitted to The Royal Children’s Hospital’s emergency department and presurgical wards, who needed intravenous maintenance hydration for 6 h or longer. Six hundred ninety participants were randomised at a 1:1 ratio to either isotonic intravenous fluid containing 140 mmol/L of sodium (Na140) or hypotonic fluid containing 77 mmol/L of sodium (Na77) for 72 h or until their intravenous fluid rate decreased to lower than 50% of the standard maintenance rate (50–150% of the daily volume recommended by [57]). Randomisation was stratified by levels of baseline sodium concentrations (Low; < 135 mmol/L, Normal; 135–145 mmol/L and High; > 145 mmol/L). The primary outcome was occurrence of hyponatraemia (defined as serum sodium concentration < 135 mmol/L with a decrease of at least 3 mmol/L from baseline) during the treatment period. A frequentist fixed trial design sample size was calculated, assuming 10% of the participants developed hyponatraemia in the Na77 group by 72 h, producing a total sample size of *n* = 640 (320 per arm) to provide 80% power with a 2-tailed 0.05 significance level to detect an absolute risk difference of 6% (calculated in nQuery [58] allowing for a continuity correction). An additional 25 participants were recruited in each arm to allow for missing data in the primary outcome, which was not incorporated into the original sample size calculation given the short time frame for the outcome.

In the original study, there were no planned interim analyses. For illustrative purposes in this tutorial, we assume that they planned to conduct a single interim analysis once half of the expected outcome events have occurred. At the interim analysis, we plan to (conservatively) declare efficacy if the *p*-value is less than 0.005. Using the traditional alpha spending framework, efficacy is declared at the final analysis if the *p*-value is less than or equal to 0.045. Given this simple design, the sample size frequentist re-calculation is *n* = 584 (292 per arm) to provide 80% power with a 2-tailed 0.045 significance level at the final analysis based on the Pearson chi-square test, to detect an absolute risk difference of 6% (equivalent to an odds ratio of 0.375). Note this is different to the original sample size calculation that used a 2-tailed 0.05 significance level. We use the design characteristics in the modified PIMS trial (with a single interim) and generate the trial data using simulation to demonstrate the expected power and sample size. Although simulation is not needed to determine the operating characteristics for this study design, we use it as an example so that we can check the results obtained from our formulaic computation above.

### Simplifying assumptions

We made the following simplifying assumptions regarding the PIMS trial: 

1. There was no loss to follow-up.
2. All sites would be active simultaneously and that the rate of recruitment would be constant, taking approximately 928 days, based on the recruitment rate in PIMS.
3. The outcome was available immediately (rather than at 72 h).
4. A single interim analysis would take place once half of the expected cases of hyponatraemia have occurred (20 cases).

**Fig. 1:**
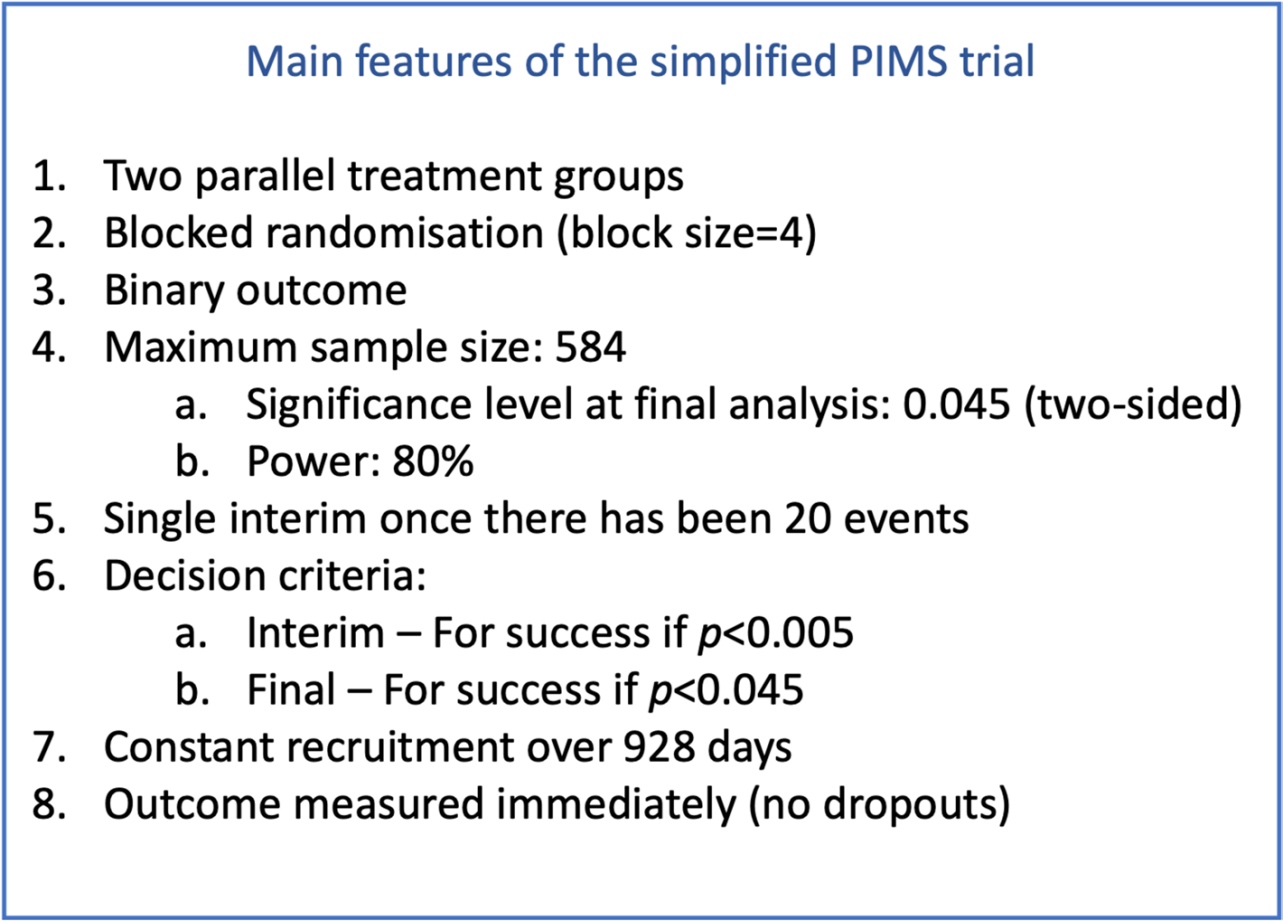
PIMS trial overview

The key features of the (modified) PIMS study design are outlined in Fig. 1.

## The simulation process

**Fig. 2:**
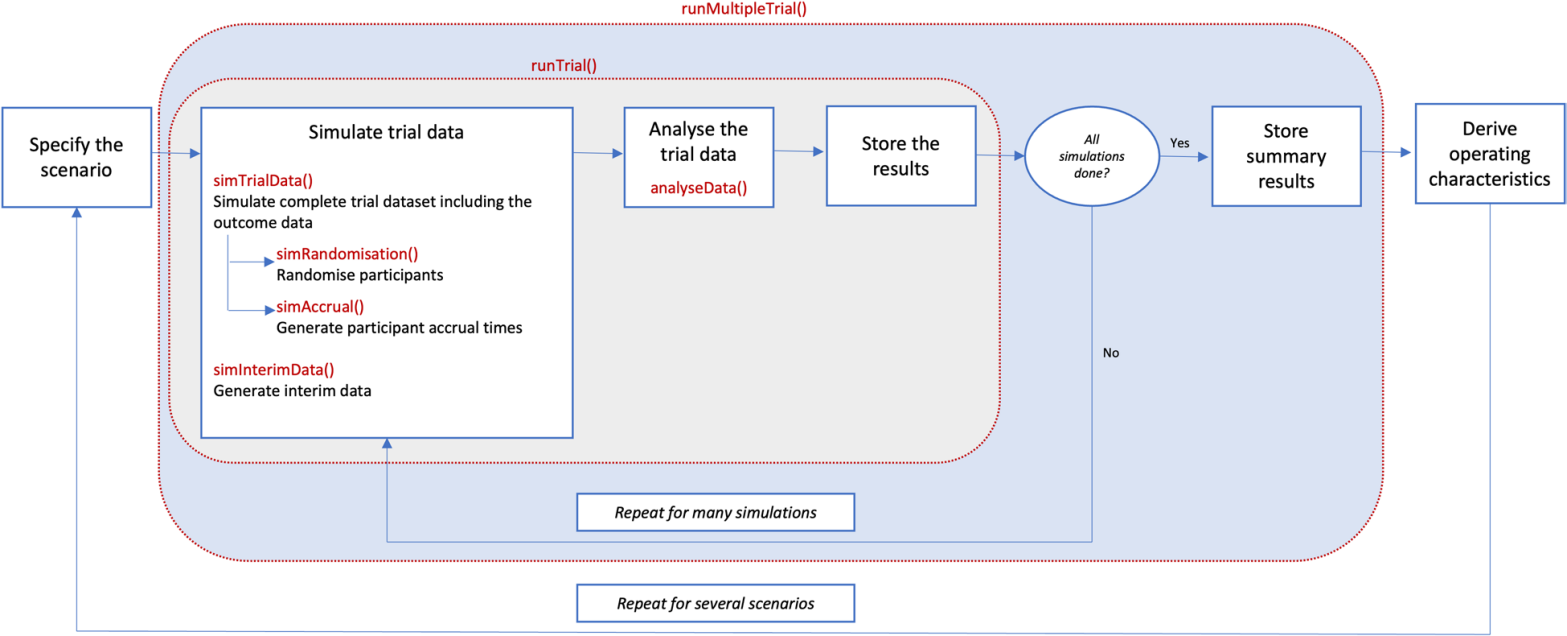
Simulation workflow and the modular structure of simulation building blocks

There are many design features to consider when planning an adaptive trial. The major considerations are the (fixed or varying) randomisation probabilities, the number and timing of interim analyses, and the decision criteria. Simulation over a range of scenarios ensures an efficient design is selected that answers the key study question(s) and balances the attitude to risk [32, 33]. Setting these design features should be an iterative procedure between clinicians and statisticians. Data for thousands of trials are simulated for a number of different scenarios (reflecting pre-determined design characteristics that align with decision points and a range of clinical effect sizes and direction of effect). It is advisable that some of the scenarios should be more extreme to determine how the trial adaptations would respond to unanticipated intervention effects. The results from these simulations are aggregated and summarised to estimate the operating characteristics under each scenario (see section “Simulation outputs”) and should be discussed with the clinical team [32, 33, 59, 60]. Once an initial set of simulation results has been obtained, the design characteristics may require adjustment, e.g., to increase the power or reduce the type I error. This process is repeated until an appropriate design with desired characteristics (such as 80% power, 5% type I error and feasible expected sample size meaningfully lower than the fixed design) has been identified. The scenarios considered should be discussed with the clinical experts and should contain a mixture of plausible and extreme scenarios reflecting various clinical effects, to provide a good understanding of how the operating characteristics change with varying treatment effects (for example different response/event rates or mean outcome in each treatment arm). This iterative procedure is outlined in Fig. 2.

When programming the simulations, it is helpful to break each trial into manageable chunks or modules that represent the stages of a trial [32]. For example, we start by generating the randomisation list, followed by recruiting participants, and then we follow them up and collect outcome data either at visits or at the end of the trial period, and then we analyse the data. We refer to the subroutines or functions that generate each stage of the trial as ‘*building blocks*’. This modular approach makes it easy to navigate through the code, enabling convenient troubleshooting, re-use and development. The code for simulation also needs to be flexible to be able to be updated with the changing trial design, as we typically want to compare multiple candidate designs with the aim of identifying an efficient design [32]. For example, a common aim of simulation is to determine the decision criteria to declare efficacy/success or futility/lack-of-benefit of the treatment(s) or trial at interim(s) and at final analysis.

In this tutorial, we generate and save the complete trial data up to the maximum recruitment and then assess the decision criteria at the interim analysis (using the available data) and the final analysis (using all of the data). The *post-processing* of the interim data means we can evaluate different decision criteria (e.g., success/futility thresholds) easily without generating the entire dataset repeatedly, provided that we have sufficient computational storage. The alternative is to assess the decision criteria once sufficient data has been generated for each interim and either continue or stop data generation depending on whether the decision threshold(s) is met. The latter approach is computationally inefficient when evaluating different decision criteria; however, it may still be needed to assess the operating characteristics of some designs such as in response adaptive randomisation [61]. Figure 2 shows a schematic of our simulation process. In the following sections, we illustrate the simulation process in R; equivalent Stata code is presented in the supplementary material.

### Building block 1: randomisation

The first step is to simulate the treatment assignment for the trial participants up to the maximum trial size. This may be via simple randomisation, blocked randomisation, stratified randomisation or more complex dynamic approaches such as minimisation [62–64]. We will focus on the most common method, block randomisation, which was employed in the PIMS trial.

Let *n* be the maximum sample size of a simulated trial, which is typically the sample size for which the study is powered to identify a clinically meaningful effect size (*n* = 584 in PIMS trial; see section “Illustrative example: the PIMS trial”) at the final analysis but is more commonly the feasible recruitment target over the trial recruitment period. The ‘*simRandomisation*’ function below generates the treatment arm allocation for “ each participant in the trial. In the PIMS trial, the participants are randomised using a 1:1 allocation ratio with block randomisation using block sizes of 4. To reflect this, we first generate blocks of size 4 (*block: 1 to 4*) and then a treatment indicator (*trt*: coded as 0 for control, i.e. Na77 group, and 1 for the Na140 group) such that two participants are allocated to each treatment arm within each block. Next, a vector of random numbers is generated from a uniform distribution between 0 and 1, and the observations are ordered by these random numbers within each block. This determines the order of treatment assignments within the block and results in a sequential list of treatment allocations for consecutively recruited participants in the trial. The input for this function is the trial maximum sample size (*n*). For simplicity, the allocation ratio of 1:1 and block size of 4 have been coded within the function. Alternatively, one could extend the function to allow the block size and the allocation probabilities to vary by including these as input variables. The output from this function is an R dataframe (dataset) with participant ID (*1:n*) and the treatment allocation (*0* or *1)* for each of the *n* participants.


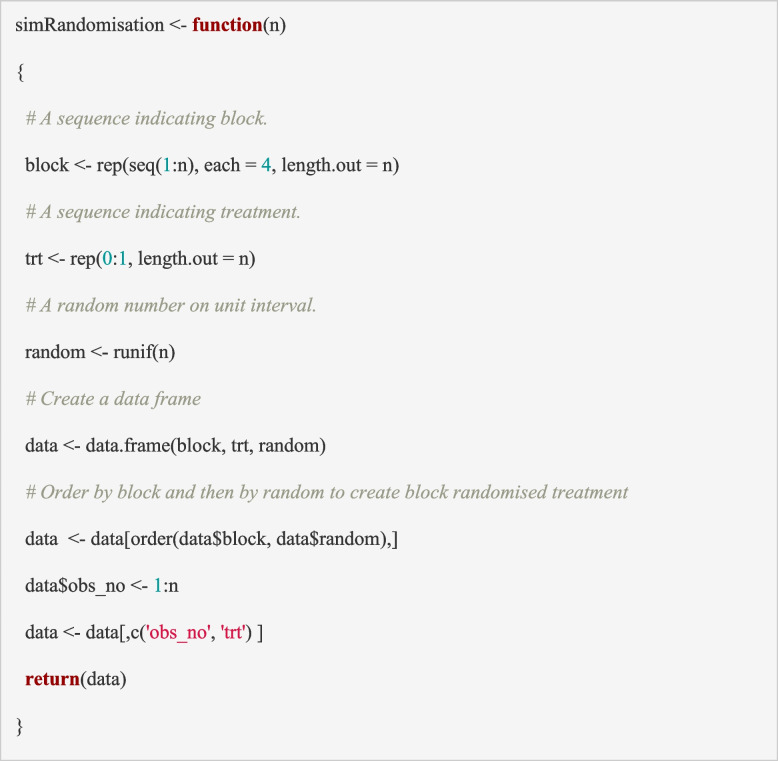


### Building block 2: simulate trial recruitment

The second step is to simulate each participant’s time of recruitment. Generating the participant accrual times should be based on a plausible recruitment rate (in days, weeks or months) across the sites. One option is to assume that participant accrual occurs at a constant rate over time from study commencement. More realistically, sites commence at different times and recruitment may ramp-up until it reaches a constant rate at which it remains until recruitment is complete. Some trials may also experience a ramp-down phase as the trial nears the end of recruitment. When simulating participant accrual, it is important to build in some variability to the recruitment process as this may affect the operating characteristics.

Participant accrual times can be generated using the function ‘*simAccrual*’. In the code below, we assume that participant accrual is constant over time and would take 928 days. The code generates *n* (the maximum sample size) random numbers from a uniform distribution between 0 and 1 and multiplies each by the length of the recruitment period (e.g., *recruit_period*: 928 days in the PIMS trial). The inputs to this function are the trial maximum sample size (*n*) and the length of the recruitment period (*recruit_period*); the output is a vector of the ordered accrual times for the *n* participants (*accrual_time*).
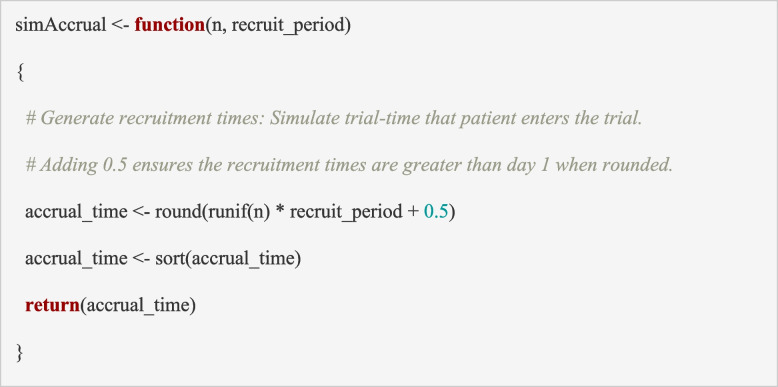


### Building block 3: generate participant outcomes

The third step is to simulate the participant outcomes under a specific scenario. Participant outcomes should be generated from the relevant probability distributions based on the outcome variable. For example, if the outcome is a binary variable (i.e., coded as 0 or 1), data can be simulated from a binomial distribution; if the outcome is a continuous variable, data can be simulated from a normal distribution; and if the outcome is a time-to-event variable, then data can be simulated from either the exponential or Weibull distribution.

In this tutorial, data are simulated using the ‘*simTrialData*’ function below, which has nested calls to the first two building blocks (‘*simRandomisation*’ and ‘*simAccrual*’). In the PIMS trial, the outcome (hyponatraemia by 72 h) is binary, and we assume that it is available for all participants immediately, hence we simulated it using a binomial distribution, with different event probabilities depending on whether the participant is allocated to the Na77 or Na140 arm (as defined in the scenarios). We define *p* as the vector of event probabilities for the two arms. Notice that the treatment allocation (*trt*) is coded as 0 for control (Na77) and 1 for treatment (Na140), therefore, when the outcome is generated, the *rbinom* function selects, the probability in vector position 0 + 1 = 1 for control and vector position 1 + 1 = 2 for treatment from vector *p*. The input to the ‘*simTrialData*’ function is the maximum sample size (*n*), the length of the recruitment period (*recruit_period*) and the vector of event probabilities (*p)*, which will depend on the scenario under consideration. The output is a dataset for a single trial with *n* rows (one for each participant) and 4 columns representing participant ID (*obs_no*), randomised treatment allocation (*trt*), accrual time (*accrual_time*) and outcome (*event*).
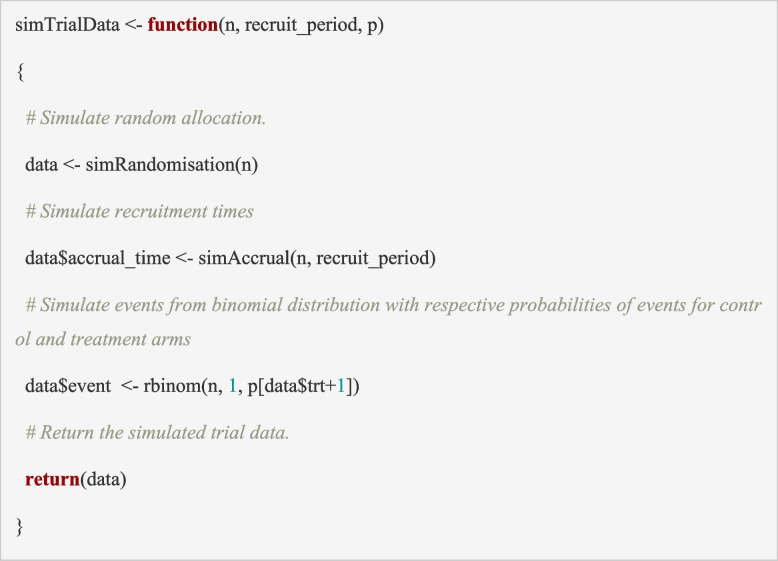


### Building block 4: identify the data available at the interim analysis

For trials that include pre-planned interim analyses, a fourth step is needed to identify and extract the data available at the time of each interim analysis. This requires identifying participants with outcomes available at the time of the interim analysis, based on their recruitment time and time to outcome, and extracting these data. When planning if and when to conduct an interim analysis, it is important to consider the time frame of the outcome relative to the recruitment period. For example, some, but not all, outcome data must be available prior to the first interim analysis. In addition, the maximum recruitment target should not be met prior to the scheduled interim analyses. Trials with a short recruitment period (e.g., weeks) relative to the time to outcome (e.g., years) are generally unsuitable for interim analyses.

The data available for an interim analysis can be identified using the function ‘*simInterimData*’. In the code, we assume that the outcome is available immediately after recruitment and the time of the interim analysis is when a pre-determined number of events (cases of hyponatraemia; *events_at_interim* = 20 events) have occurred. Since the data are ordered by participant recruitment times (see section ‘Building block 2: simulate trial recruitment’), we can compute the cumulative number of events (*cum_events)* using a running total of the column containing the outcome data and the participant ID (*obs_no*) at which 20 events are accumulated (i.e., when *cum_events* = 20*)* indicates the planned time of our interim analysis (*interim_ind*). In this example, as there is no time lag between recruitment and outcome assessment, the outcomes at the interim (*event_interim*: 0 or 1) would be the same as the outcomes at the final timepoint (*event*) for participants included in the interim analysis (i.e., for observations where *obs_no* < = *interim_ind*).

The input to this function is the simulated trial dataset (*data*) and the number of events triggering the interim analysis (*events_at_interim*) and the output is the trial dataset with additional columns for the data included in the interim analysis (includes *cum_events, interim_ind, event_interim*). Note that the *event_interim* variable has missing values for all the participants recruited after the interim timepoint. These participants will be excluded from the interim analysis (see section ‘Building block 5: analyse the trial data’). Alternatively, the user may choose to only extract the data up to the interim timepoint and output it as a separate truncated dataset (*data_interim;* shown within the comments of code below) and then use this dataset as an input to the interim analysis function (section ‘Building block 5: analyse the trial data’). The ‘*simInterimData*’ function can be modified to reflect multiple interims performed when a fixed number of new participants have accrued (e.g., every 20) and to allow a lag time between recruitment and outcome assessment (e.g., outcome at 2 weeks).


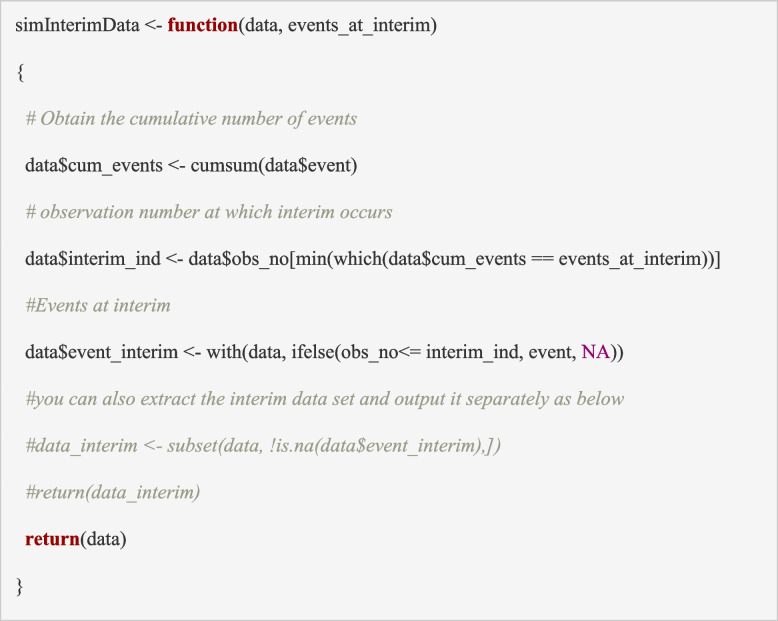


### Building block 5: analyse the trial data

The fifth and final step is to conduct the analysis of the trial data. This function is generic and can be used for the analysis at an interim and at the end of the study. Generally, only the primary outcome is analysed to compare the treatments against the control, based on participants with available data up to that timepoint. The test statistics are evaluated against decision criteria to determine which treatment arms will continue to have new participants assigned to them, which treatment arms will have no further new assignments (i.e., arm dropped at interim), and whether the trial has reached a conclusion that triggers the final analysis (which would include the analysis of all secondary endpoints). In the modified PIMS trial, whether or not a simulated trial would have stopped recruitment (due to superiority of the treatment arm over control) at an interim analysis is assessed by comparing the test statistics against the pre-defined stopping boundaries, i.e., evaluating whether the interim *p*-value is less than 0.005. Some quantities that can be useful to output from the analysis are:

1. Whether the trial would have stopped before maximum recruitment at each interim analysis
2. Point estimate and confidence interval for the treatment effect (for example, odds ratio or relative risk) at the final analysis (which may be at the interim timepoint if the study was stopped early).
3. The sample size at the time the trial was stopped (including when maximum recruitment was reached).
4. Whether the study would have found evidence of clinically relevant treatment effects or if the trial was inconclusive.

The function ‘*analyseData*’ can be used to analyse the data for each trial at each scheduled analysis and evaluate decision criteria for adaptations. It calculates the test statistic using a statistical model (in the PIMS trial, it is a logistic regression model) for the statistical hypothesis being explored, e.g., whether treatment is superior to control. It then compares the test statistics against the pre-defined decision threshold and determines whether the criterion for stopping recruitment at the interim timepoint has been met, in addition to whether the trial conclusion is reached before maximum recruitment. The results from each analysis, such as the estimate of the effect size and associated confidence interval and whether decision thresholds are met at interim(s) and final analysis, are saved as the output.

Specifically, the following steps are carried out in the ‘*analyseData*’ function:

1. Compute the proportion of participants with an event in the control (*pevents0*) and treatment (*pevents1*) arms at maximum recruitment (end of the trial) or at the interim if the trial stopped early.
2. Conduct a logistic regression to compare outcomes between the treatment and control arms at the interim analysis.
3. Assess the decision criteria at the interim time point, i.e., is the *p*-value for the log odds ratio for treatment compared to control *(interim_p)* less than the decision threshold at the interim (*alpha_interim*). If true, then stop recruitment to the trial at the interim and declare efficacy/success (i.e., *interim_stop* = 1), otherwise continue recruitment.
4. Conduct a logistic regression to compare outcomes between the treatment and control arms at the final analysis.
5. Assess the decision criteria at the final analysis and declare efficacy/success if the *p*-value for the log odds ratio for treatment compared to control (*final_p)* is less than the decision threshold at the final analysis (*alpha_final*), otherwise declare futility.
6. Record the trial conclusion in the variable *final_stop*, where *final_stop* = 1 if the treatment was determined to be efficacious compared to control, or *final_stop* = 0 otherwise.

The inputs to this function are the simulated dataset from ‘*siminterimData*’ (*data*) and the decision thresholds at each time point (*alpha_interim* and *alpha_final*). The decision thresholds are usually chosen by simulation to control the false-positive error and should be pre-specified in the trial protocol. Users may be interested in exploring different thresholds as part of the simulation exercise. The output is a summary of the results from the interim and final analyses (*results*), including the number and proportion of events in each treatment arm (*nevents0, nevents1, pevents0, pevents1*), the sample size (*sample_size:* which is either the number of participants recruited at the interim if the trial stopped early or the maximum sample size *n,* otherwise), the time of the interim (*interim_time*), the effect sizes (odds ratios) and confidence intervals at the interim and at the final analysis (*interim_or, interim_lci, interim_uci, final_or, final_lci, final_uci*), the *p*-values at the interim and final analysis (*interim_p, final_p*), whether the trial reached an efficacy conclusion at the interim and final time points (*interim_stop, final_stop*), whether the trial was conclusive (*stop*: 1, if trial met the decision threshold at the interim or final analysis, or 0, otherwise) and the probability of trial flip-flopping (*flipflop*: 1, if the trial met the decision threshold at the interim but not at the final analysis, or 0, otherwise).


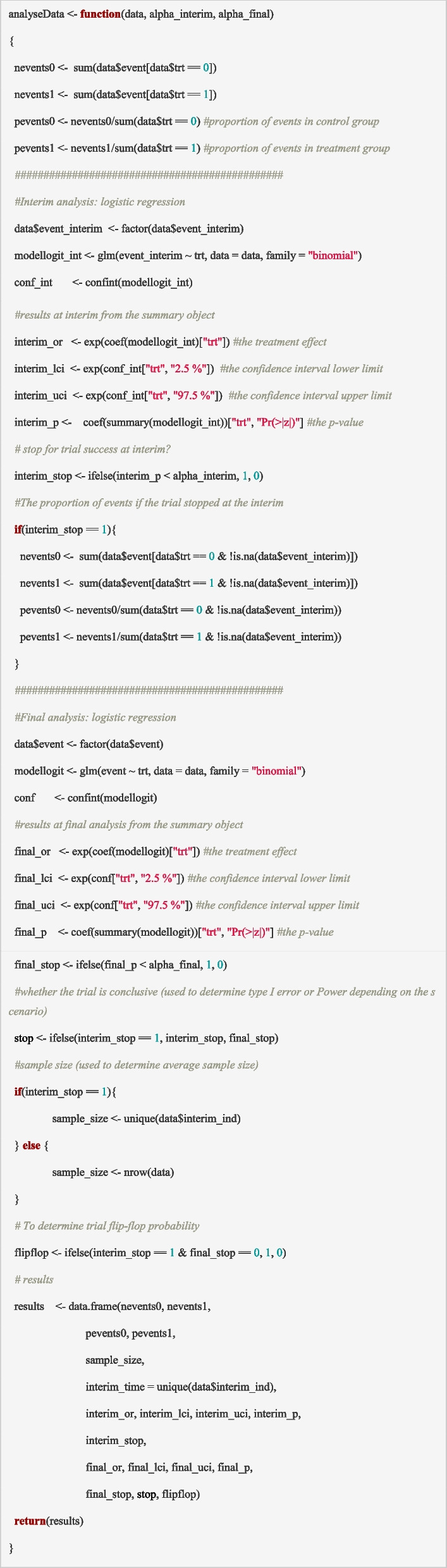


### Simulating a single trial

The building blocks above (functions in sections ‘Building block 3: generate participant outcomes’, ‘Building block 4: identify the data available at the interim analysis’, to ‘Building block 5: analyse the trial data’) can be put together to conduct the simulations for a single trial. We begin by simulating a single trial which assists in debugging the code and identifying whether all relevant results have been captured. We define a number of global parameters (which represent the simulation inputs) described below, and then sequentially run each step using the ‘*runTrial*’ function.

## Inputs

In order to simulate trial data, we must specify a number of global parameters to use in our simulation. Below we outline the global parameters we use for the PIMS trial:

1. The random seed to ensure reproducibility of the data and outputs.
2. The recruitment period, which for the PIMS trial we assumed to be 928 days.
3. The maximum trial sample size (n = 584 for PIMS trial).
4. The number of events required to trigger an interim analysis (20 cases of hyponatraemia in PIMS trial).
5. The proportion with the event in the treatment and control arms. This is expressed as a vector, where the values depend on the scenario for which data is being generated. Initially we set these as p0 = 0.10 and p1 = 0.04 which we denote as the ‘as powered’ scenario.
6. The decision thresholds, which were set to match the fixed-design sample size calculation, i.e., 0.005 at the interim and 0.045 at the final analysis (*alpha_interim* = 0.005, *alpha_final* = 0.045).

It is useful to define the input parameters in one place so that this list can be easily accessed for reference at any time and can be updated to explore alternative designs or scenarios. In the PIMS example, we use the following code to detail the inputs.


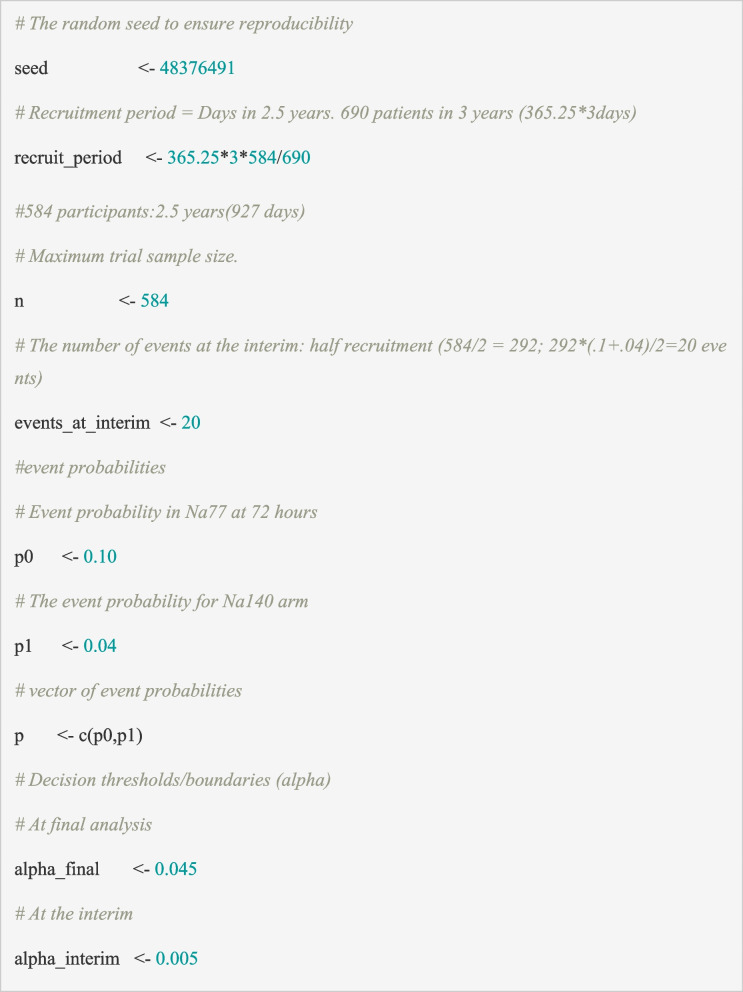


The function ‘runTrial’ below uses the previously defined building blocks to simulate data for a single trial:

1. Building block 3: ‘*simTrialData*’ simulates the trial data (calls ‘*simRandomisation’ and ‘simAccrual*’)
2. Building block 4: ‘*simInterimData*’ identifies the data for the interim analysis
3. Building block 5: ‘*analyseData*’ analyses the trial data

The inputs are the maximum sample size (*n*), the recruitment period (*recruit_period)*, the vector of event proportions in the treatment arms (*p*), the number of events to trigger the interim (*events_at_interim*) and the decision thresholds at the interim(s) and final analysis (*alpha_interim* and *alpha_final,* respectively). The output is a list containing the simulated dataset (*data*) and the results from the analyses of interim and final data (*results*).


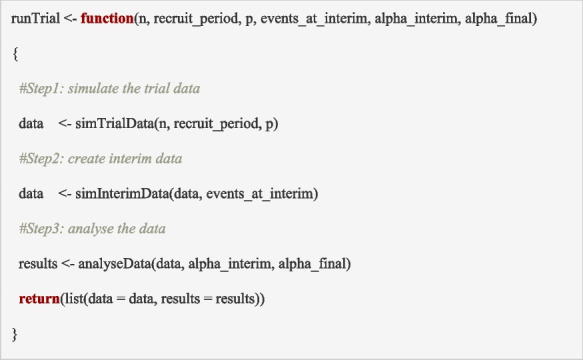


This function can be executed to generate the trial data for a single trial using the following code:


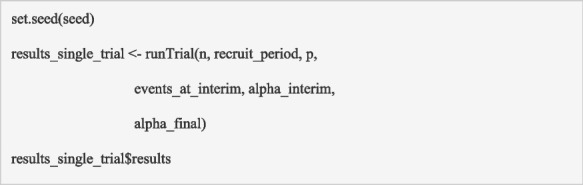


The output generated from this function is illustrated in Table 1. The output includes the simulated dataset, the results from the analyses and the evaluation of the decision criteria. The results (contained within *results_single_trial$results*) includes the variables described in section ‘Building block 5: analyse the trial data’ (output from the function ‘*analyseData*’).

**Table 1:** The output from a single simulated trial dataset

Variable	Description of the variable	Output
nevents0	Number of events in the control group	17
nevents1	Number of events in the treatment group	9
pevents0	Proportion of events in the control group	0.06
pevents1	Proportion of events in the treatment group	0.03
sample_size	Sample size	584
interim_time	Time at the interim (days)	433
interim_or	Odds ratio at the interim	0.81
interim_lci	Lower CI for OR at interim	0.32
interim_uci	Upper CI for OR at interim	2.01
interim_p	P-value for any difference between treatments at the interim analysis	0.66
interim_stop	Whether the trial would have stopped at the interim (based on decision criteria at interim)	0.00
final_or	Odds ratio at final analysis	0.51
final_lci	Lower CI for odds ratio	0.22
final_uci	Upper CI for odds ratio	1.15
final_p	P-value for treatment difference at final analysis	0.11
final_stop	Whether the trial is conclusive at final analysis	0.00
stop	Whether the trial was conclusive (at the interim or at the final analysis)	0.00
flipflop	The probability of trial flip-flopping	0.00

The control group (Na77); the treatment group (Na140)

### Simulating multiple trials

The ‘*runTrial*’ function simulates data for a single trial. However, a single trial is not representative of what to expect for a particular scenario, i.e., some simulated trials will have more extreme intervention effects than others. It is therefore important to simulate many trials for each scenario of interest to understand how our trial design could plausibly perform accounting for the variability in the trial. To do this, we create a function, ‘*runMultipleTrials*’, that repeatedly executes the ‘*runTrial*’ function and saves the summary for each trial. Note that we can save all the simulated trials/datasets (using ‘*saveRDS*’ in the function below). This may take up a considerable amount of space depending on the number of simulations; however, it can be useful if additional summary measures may be required in the future.

The inputs required for the ‘*runMultipleTrials*’ function (in addition to the global parameters) are the random seed (*seed*) and the number of trial data sets to be simulated (*simno*). Note that setting the seed once before running any of these functions will make the results reproducible. However, in this implementation we have used seed within the function to make it explicit and part of the function, so that the code can be executed in isolation. The output of this function is a list containing a data frame of the results as returned by the ‘*analyseData*’ function for each of the trial datasets simulated (*results_all*), a data frame with the statistical summaries of the results across all of the simulated trials (*results_summary*) and the seeds used for reproducibility (*seeds*).
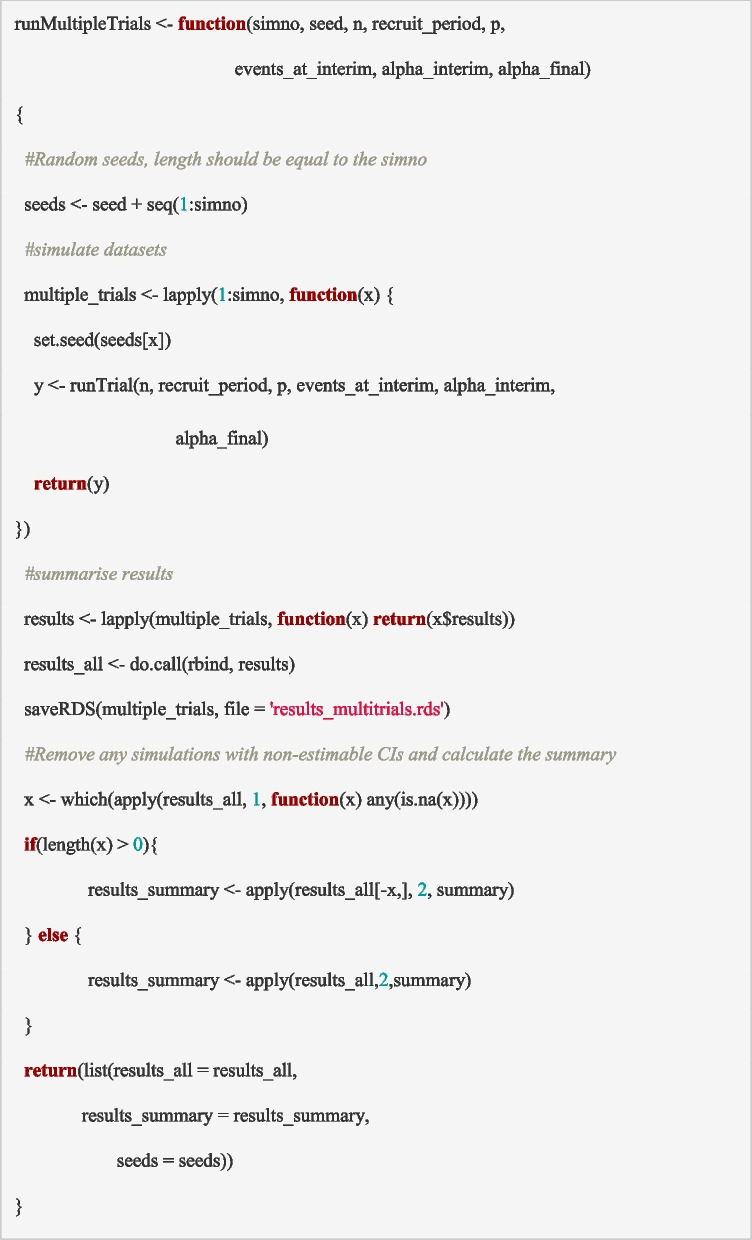


For the PIMS trial, we generated 5000 simulated datasets (*simno* = 5000) for each scenario, which is appropriate for the expected accuracy of the summary measures across simulations (larger numbers tend to give more accurate estimates) given the computational burden. Simulation of more complex trials may need larger numbers. Practically, it can be useful to start with a much smaller number of simulations (e.g., *simno* = 10 or 100) to ensure that the function is working as expected, before increasing to a larger number to compare the choice of design parameters. The ‘*runMultipleTrials*’ function can be executed using the code below.


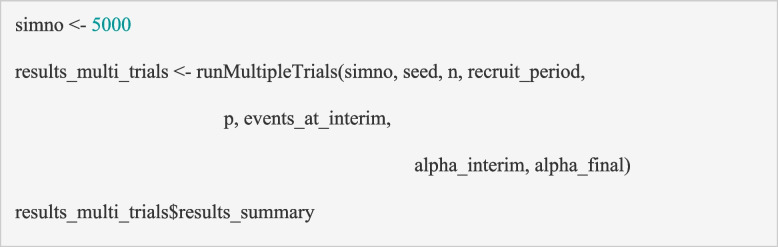


The summary output from this function is presented in Table 2 for the ‘as powered’ scenario. It includes the statistical summaries (minimum, 1st quartile, median, mean, 3rd quartile and maximum) of all of the output variables in the ‘*results*’ dataset from the ‘*analyseData*’ function (see section ‘Building block 5: analyse the trial data’) across the 5000 simulated trials (e.g., mean sample size for the 5000 simulated trials).

**Table 2:** A summary of the output from 5,000 simulated trial datasets for the expected treatment effect in PIMS (p0 = 0.10 for control arm (Na77), p1 = 0.04 for treatment arm (Na140))

Variable	Description of the variable	Minimum	Q1	Median	Mean	Q3	Maximum
nevents0	Number of events in the control group	14	23	28	27	31	49
nevents1	Number of events in the treatment group	1	9	11	11	14	24
pevents0	Proportion of events in the control group	0.05	0.09	0.10	0.10	0.11	0.27
pevents1	Proportion of events in the treatment group	0.01	0.03	0.04	0.04	0.05	0.08
sample_size	Sample size	129	584	584	**545**	584	584
interim_time	Time at the interim (days)	116	240	281	284	324	548
interim_or	Odds ratio at the interim	0.04	0.23	0.40	0.41	0.52	1.95
interim_lci	Lower CI for OR at interim	0.00	0.07	0.14	0.14	0.19	0.78
interim_uci	Upper CI for OR at interim	0.21	0.65	1.03	1.06	1.30	5.31
interim_p	P-value for any difference between treatments at the interim analysis	0.00	0.01	0.07	0.14	0.17	1.00
interim_stop	Whether the trial would have stopped at the interim (based on decision criteria at interim)	0.00	0.00	0.00	**0.13**	0.00	1.00
final_or	Odds ratio at final analysis	0.06	0.29	0.37	**0.39**	0.48	1.24
final_lci	Lower CI for odds ratio	0.01	0.13	0.18	**0.19**	0.24	0.65
final_uci	Upper CI for odds ratio	0.19	0.58	0.74	**0.77**	0.91	2.41
final_p	P-value for treatment difference at final analysis	0.00	0.00	0.01	**0.04**	0.03	1.00
final_stop	Whether the trial is conclusive at final analysis	0.00	1.00	1.00	0.80	1.00	1.00
stop	Whether the trial was conclusive (at the interim or at the final analysis)	0.00	1.00	1.00	**0.80**	1.00	1.00
flipflop	The probability of trial flip-flopping	0.000	0.000	0.000	**0.001**	0.000	1.000

Q1: 1st quartile; Q3: 3rd quartile. In bold are the quantities discussed in the manuscript. Namely, the average sample size, probability of trial stopping early (at interim), average of final odds ratio and confidence interval [CI] (lower CI boundary [LCI], upper CI boundary [UCI]), *p*-value, probability of trial success, probability of trial flipflopping (rounded to 3 decimal places due to small magnitude)

## Simulation outputs

### Scenarios

As discussed previously, trial simulation involves evaluating the trial operating characteristics for a range of different scenarios [59]. Most of these scenarios should be based on plausible quantities for effect sizes between treatment arms according to expert opinion and pilot studies. However, it is important to consider some extreme scenarios to develop a good understanding of how the trial might perform if these extreme scenarios arise in practice, such as if effect sizes were much larger or much smaller than current evidence. For the PIMS trial, the scenarios that could be considered are outlined in Table 3. The ‘null’ scenario represents the scenario where there is no difference in the primary outcome between Na140 and the Na77 groups, and the ‘as powered’ scenario represents the scenario used in the original fixed-design sample size calculation. We have also considered two ‘extreme’ scenarios where the treatment effect is smaller and larger than the expected effect.

**Table 3:** Event probabilities across treatment arms for the 4 scenarios considered in the PIMS simulations

Scenario	Control (Na77)	Intervention (Na140)
Null	0.10	0.10
As powered	0.10	0.04
Smaller difference	0.10	0.06
Larger difference	0.10	0.03

To evaluate the trial operating characteristics for each of the scenarios we use the function ‘*runMultipleTrials*’ changing the input parameters regarding the primary outcome. We demonstrated the ‘as powered’ in Section "Simulating multiple trials" above (p0 = 0.10 for control arm, p1 = 0.04 for treatment arm). In Table 4 and Supplementary Tables 1 and 2 we present the summary of the results across 5000 simulated trials for ‘the null hypothesis’ scenario, ‘smaller difference’ scenario and ‘larger difference’ scenario respectively.

**Table 4:** A summary of the output from 5000 simulated trial datasets under the ‘null hypothesis’ scenario for the PIMS simulations

Variable	Description of the variable	Minimum	Q1	Median	Mean	Q3	Maximum
nevents0	Number of events in the control group	2	26	29	29	33	49
nevents1	Number of events in the treatment group	2	26	29	29	33	47
pevents0	Proportion of events in the control group	0.03	0.09	0.10	0.10	0.11	0.23
pevents1	Proportion of events in the treatment group	0.02	0.09	0.10	0.10	0.11	0.25
sample_size	Sample size	141	584	584	583	584	584
interim_time	Time at the interim (days)	80	169	197	199	227	378
interim_or	Odds ratio at the interim	0.10	0.78	1.00	1.12	1.26	11.55
interim_lci	Lower CI for OR at interim	0.02	0.30	0.39	0.43	0.50	3.15
interim_uci	Upper CI for OR at interim	0.34	2.00	2.55	2.99	3.31	74.69
interim_p	*P*-value for any difference between treatments at the interim analysis	0.00	0.33	0.62	0.52	0.66	1.00
interim_stop	Whether the trial would have stopped at the interim (based on decision criteria at interim)	0.00	0.00	0.00	0.00	0.00	1.00
final_or	Odds ratio at final analysis	0.36	0.83	1.00	1.04	1.21	2.74
final_lci	Lower CI for odds ratio	0.19	0.48	0.58	0.60	0.70	1.52
final_uci	Upper CI for odds ratio	0.66	1.43	1.73	1.81	2.10	5.15
final_p	*P*-value for treatment difference at final analysis	0.00	0.25	0.49	0.50	0.77	1.00
final_stop	Whether the trial is conclusive at final analysis	0.00	0.00	0.00	0.04	0.00	1.00
stop	Whether the trial was conclusive (at the interim or at the final analysis)	0.000	0.000	0.000	**0.043**	0.000	1.000
flipflop	The probability of trial flip-flopping	0.000	0.000	0.000	0.001	0.000	1.000

Q1: 1st quartile; Q3: 3rd quartile. In bold is the proportion of trials that conclude as a success when there is no treatment effect (i.e., type I error)

### Interpreting the output

Once we have run many simulations per scenario, we use the summary measures from these scenarios to tell us about the operating characteristics of the design as described below. Example code to obtain the operating characteristics is given in the Supplementary material.

#### Operating characteristics of interest

##### Probability of trial success when there is no treatment difference (type I error)

One of the key operating characteristics is the type I error. The type I error is the probability of rejecting the null hypothesis (i.e., declaring a trial success or identifying a treatment effect) when there is no treatment effect. We often aim to control the type I error to be below 5%. From our simulation outputs, the type I error is estimated by the proportion of trials that conclude as a success (i.e. declared a difference between treatment arms) in the ‘null hypothesis’ scenario, where there truly was no difference between the treatment and control arms [11]. The mean value of the *stop* variable (whether the trial was conclusive at the interim or final analysis) across all simulated datasets under the ‘null hypothesis’ scenario provides an estimate for the type I error.

##### Probability of trial success when the intervention is truly superior (power)

Another important operating characteristic to consider is the power of the trial. The power of the trial to detect a treatment effect is reflected in the proportion of successful trials (i.e. those that declare a difference between treatment arms) where there truly is a difference between the intervention arms. For example, the power of the trial to detect the treatment effect in the original sample size calculation of the PIMS trial can be estimated using the mean value of the *stop* variable in the ‘as powered’ scenario. That is, the proportion of trials that conclude the treatment arm is superior to the control arm at the interim or final analysis in the ‘as powered’ scenario.

##### Probability of stopping at the interim analysis

The probability of the trial stopping at the interim analysis due to reaching a decision threshold is another operating characteristic of interest. This is calculated from the mean value of *interim_stop* variable across simulated trials for a given scenario.

##### Mean number of participants per trial

The mean sample size (*sample_size*) can be used to assess average reduction in trial size due to the inclusion of interim analyses. This gives an indication of the usefulness of the interim analysis(es), if we can save both time and resources without recruiting further participants or collecting further follow-up data.

##### Probability of the trial ‘flip-flopping’

The probability of a ‘flip-flop’ is another characteristic that may be of interest to explore in the simulation output. This occurs when a given simulated trial is flagged as reaching a decision threshold at an interim analysis, but the critical value for declaring a difference at the final analysis is not met. This is also known as the ‘false stopping probability’, where we would stop the trial at the interim analysis for success or futility (*interim_stop* = *1*); however, if we had continued the trial until final analysis this decision threshold would not have been reached (*final_stop* = *0*) [65]. The trial should be designed such that this probability is small. Often this probability can be minimised by the choice of decision threshold [11]. The probability of a flip-flop can be obtained using the mean value of the *flipflop* variable in the output.

##### Estimated treatment effects (is the model doing its job?)

A final output that may be of interest is the estimated treatment effect(s) and the confidence intervals from the final analysis or at the interim analysis if the trial stops at the interim. This output can be useful to check model bias and to ensure that these quantities reflect the true values we used in the simulation.

## Outputs from PIMS

In the PIMS trial, the proportion of trials that conclude the intervention is superior to control in the null scenario (i.e., type I error) is 0.043 (Table 4). This reflects the alpha value (type 1 error) for the final analysis that was used in the modified sample size calculation (alpha = 0.045). The probability of trial success under the ‘as powered’ scenario is 0.8 (Table 2), which reflects the 80% power obtained for this treatment effect in the modified sample size calculation. The probability of the trial stopping at the interim analysis for efficacy in the ‘as powered’ scenario is 0.13 (Table 2). Under this scenario, the average sample size is 545, which is slightly lower than the maximum sample size of 584 from the modified sample size calculation as expected. The mean estimates of the odds ratio at the final analysis (*final_or*), its confidence interval (*final_lci, final_uci*) and the *p*-value for the difference between the two treatment groups (*final_p*) are 0.39 [0.19, 0.77], *p* = 0.04 (Table 2). This is close to the odds ratio of 0.375 used in the modified sample size calculation. The probability of trial flip-flopping is 0.001.

## Discussion

Adaptive trials are gaining popularity due to their flexibility and efficiency [6, 66]. When designing adaptive trials, simulation is often required to select the most appropriate design, explore the trial operating characteristics, and determine the expected sample size. Simulation requires statistical programming skills that involve data generation, manipulation and generating appropriate summaries. It can be computationally intensive due to the range of design parameters and assumptions to be explored (e.g., effect sizes, decision criteria, number and timing of interim analyses, maximum sample sizes) and the potentially large number of scenarios to explore [32, 33, 59].

In this tutorial, we have shown how to simulate an adaptive trial and provided example code in R and Stata. For simplicity, we focused on a simple parallel-group study with a single interim analysis, where the operating characteristics were known so that we can replicate the results in the simulations. In practice, the operating characteristics are unknown and cannot be simply derived without the use of sometimes complex simulation. The simulation process often involves numerous iterations of setting the design features/parameters and running simulations across a range of potential scenarios [32, 33, 59]. This is generally through a feedback loop between the clinical and the statistical teams, where initially the scenarios are defined based on historical or pilot data from the clinical team and the inputs to the functions are updated based on the output from previous simulation runs. This process is repeated until desirable statistical properties are achieved across all plausible scenarios and risk is assessed for unexpected scenarios, thus determining an efficient trial design. This tutorial serves as a practical resource aimed at improving the accessibility of the simulation process for both statisticians and clinicians.

The simulation process has been described in a previous tutorial by Hansen et. al. [32], although this previous tutorial focussed on the use of BUGS, a Bayesian programme language that may not be familiar to most statisticians and clinicians, and the implementation of the coding rather than the full design process, including review cycles that use the results from successive simulations to hone in on an efficient trial design. Our tutorial extends previous work by providing implementation code in R and Stata—two widely used statistical packages—and offering structured guidance on presenting and interpreting simulation results. This approach is intended to enhance accessibility for both statisticians and clinicians.

The modular coding structure we have used in our tutorial (Fig. 2) also makes our approach appealing, as it makes it easy to troubleshoot and modify aspects of the code without having to amend the full code. It also provides the flexibility of exploring many scenarios and design parameters using the same set of building blocks. When conducting simulations for guiding study planning, the code should be written in a way that can be used and modified for multiple scenarios and design characteristics efficiently. It is also important to ensure computational efficiency as simulating complex adaptive designs are much more time consuming than standard trials. If you have access to multiple Central Processing Units (CPUs), efficiency can be improved by running several R sessions in parallel. We have provided an example of the use of parallel processing for the simulation in the hope of improving computational efficiency within the supplementary materials.

When simulating data for a particular trial design, we recommend starting by simulating a single trial and exploring the results to identify any errors in the codes, and whether the desired results are stored appropriately. As a second step, multiple trials should be simulated initially simulating 5–10 trials to check the summaries across the simulated trials, before simulating a large number (over 1000) of trials. This staged process ensures that once a large number of simulations are being run, the analyst has confidence in the results. The output from a single trial can also be used as a training tool for Data Safety and Monitoring Committees (DSMC’s), especially when the trial is complex. A review of the interim results from selected trial simulations can also provide good examples to the DSMC on what may happen during the trial.

In this tutorial, we illustrated the simulation process and code using the PIMS trial, however, these building blocks can be adapted and expanded for other studies. We have included R code within the manuscript and equivalent Stata code can be found in the supplementary material. In practice, designing an adaptive trial is often more complicated than the example presented here, and the features of the design will need to be incorporated into the simulation code.

## Conclusion

Trial simulation is typically required for resource planning for adaptive designs, which must be tailored to the research questions, features and requirements of the trial at hand. In this tutorial, we provide researchers with the building blocks to conduct such simulations that are accessible to statisticians and clinical trialists and can be tailored to suit their study needs.

## Supplementary Information

Supplementary Material 1

## Data Availability

Not applicable.
