# Supplementary material for: A practical guide to simulation for an adaptive trial design with a single interim analysis
Source: Trials. 2025 Oct 9;26:400. doi: 10.1186/s13063-025-09100-5 (PMC12512321; doi:10.1186/s13063-025-09100-5)
Supplement: Supplementary file 1 — Supplementary Material 1 [file 13063_2025_9100_MOESM1_ESM.docx]

# Supplementary material

## Simulating multiple trials using parallel processing

The code in section 3.7 (within the main manuscript) can take a long time to run if you need a large number of simulations. If you have access to multiple Central Processing Units (CPUs), efficiency can be improved by running several R sessions in parallel. There can be multiple ways to implement parallel processing within the R environment. We demonstrate below, two such implementations of parallel processing using ‘doParallel’ and ‘parallel’ R packages. You can specify the number of cores to be used for this process; we have used one less than the total available number of cores in the computer (*detectCores()-1*).

# Implementing parallel processing via ‘doParallel’ R package.

runMultipleTrialsParallel <- **function**(simno, seed, n, recruit_period, p,

events_at_interim, alpha_interim,

alpha_final)

{

*#set-up for parallel processing*

**library**(foreach)

**library**(doParallel)

**library**(snow)

cl <- makeCluster(parallel::detectCores()-1, type = "SOCK")

clusterExport(cl, c("runTrial","analyseData","simTrialData",

"simInterimData","simRandomisation","simAccrual"),

envir = environment())

registerDoParallel(cl) *#use (no: of available cores -1 )*

seeds <- seed+seq(1:simno) *#Random seeds, length equal to the simno*

*##########################################################################*

*#simulate datasets*

multiple_trials <- foreach(x=1:simno) %dopar% {

set.seed(seeds[x])

y <- runTrial(n, recruit_period, p, events_at_interim, alpha_interim,

alpha_final)

**return**(y)

}

*#summarise results*

results <- lapply(multiple_trials,**function**(x)**return**(x$results))

results_all <- do.call(rbind,results)

saveRDS(multiple_trials,file='results_multitrials.rds')

*#Remove any simulations with non estimable CIs and calculate the summary*

x <- which(apply(results_all,1,**function**(x)any(is.na(x))))

**if**(length(x)>0){results_summary <- apply(results_all[-x,],2,summary)

}**else**{results_summary <- apply(results_all,2,summary)}

**return**(list(results_all=results_all, results_summary=results_summary, seeds=seeds))

}

The time taken to generate the simulated data changes according to the processing speed of the computer, the number of cores used and the number of simulations. To illustrate the utility of ‘*runMultipleTrialsParallel’* function, we compared the execution times for this function and the ‘*runMultipleTrials’* function without parallel processing, when run on the same computer for 1,000 simulations. In this instance, the processing time is reduced to more than a third of the original (15 vs. 5 seconds). We acknowledge that for a relatively simple adaptive trial such as the one presented in this tutorial, parallel processing may not add much value in terms of efficiency. However, adaptive trials are much more complex in reality and hence it is hoped that the above code will help the user in such situations.

# Implementing parallel processing via ‘parallel’ R package.

Below we present the implementation of parallel processing using ‘parallel’ R package.

runMultipleTrialsParallel <- **function**(simno, seed, n, recruit_period, p, events_at_interim, alpha_interim,

alpha_final)

{

seeds <- seed+seq(1:simno) *#Random seeds, length equal to the simno*

*#parallel processing with mclapply*

**library**(parallel)

*#simulate datasets*

multiple_trials <- mclapply(1:simno, **function**(x){

set.seed(seeds[x])

y <- runTrial(n, recruit_period, p, events_at_interim, alpha_interim, alpha_final)

**return**(y)

}, mc.cores = detectCores()-1)

*#summarise results*

results <- lapply(multiple_trials,**function**(x)**return**(x$results))

results_all <- do.call(rbind,results)

saveRDS(multiple_trials,file='results_multitrials.rds')

*#Remove any simulations with non estimable CIs and calculate the summary*

x <- which(apply(results_all,1,**function**(x)any(is.na(x))))

**if**(length(x)>0){results_summary <- apply(results_all[-x,],2,summary)

}**else**{results_summary <- apply(results_all,2,summary)}

**return**(list(results_all=results_all, results_summary=results_summary, seeds=seeds))

}

# Results of simulations under the ‘smaller difference’ scenario.

Supplementary Table 1. A summary of the output from 5,000 simulated trial datasets under the ‘smaller difference’ scenario.

| Variable | Description of the variable | Minimum | Q1 | Median | Mean | Q3 | Maximum |
| --- | --- | --- | --- | --- | --- | --- | --- |
| nevents0 | Number of events in the control group | 14 | 25 | 29 | 29 | 32 | 49 |
| nevents1 | Number of events in the treatment group | 1 | 15 | 17 | 17 | 20 | 34 |
| pevents0 | Proportion of events in the control group | 0.05 | 0.09 | 0.10 | 0.10 | 0.11 | 0.27 |
| pevents1 | Proportion of events in the treatment group | 0.01 | 0.05 | 0.06 | 0.06 | 0.07 | 0.12 |
| sample_size | Sample size | 135 | 584 | 584 | 577 | 584 | 584 |
| interim_time | Time at the interim (days) | 108 | 211 | 245 | 249 | 284 | 445 |
| interim_or | Odds ratio at the interim | 0.04 | 0.40 | 0.52 | 0.62 | 0.80 | 4.73 |
| interim_lci | Lower CI for OR at interim | 0.00 | 0.14 | 0.19 | 0.23 | 0.31 | 1.65 |
| interim_uci | Upper CI for OR at interim | 0.22 | 1.04 | 1.31 | 1.60 | 2.01 | 17.10 |
| interim_p | P-value for any difference between treatments at the interim analysis | 0.00 | 0.07 | 0.18 | 0.32 | 0.63 | 1.00 |
| interim_stop | Whether the trial would have stopped at the interim (based on decision criteria at interim) | 0.00 | 0.00 | 0.00 | 0.02 | 0.00 | 1.00 |
| final_or | Odds ratio at final analysis | 0.17 | 0.46 | 0.57 | 0.60 | 0.70 | 1.62 |
| final_lci | Lower CI for odds ratio | 0.07 | 0.24 | 0.30 | 0.32 | 0.38 | 0.88 |
| final_uci | Upper CI for odds ratio | 0.36 | 0.85 | 1.04 | 1.11 | 1.31 | 3.15 |
| final_p | P-value for treatment difference at final analysis | 0.00 | 0.02 | 0.07 | 0.19 | 0.26 | 1.00 |
| final_stop | Whether the trial is conclusive at final analysis | 0.00 | 0.00 | 0.00 | 0.40 | 1.00 | 1.00 |
| stop | Whether the trial was conclusive (at the interim or at the final analysis) | 0.00 | 0.00 | 0.00 | 0.40 | 1.00 | 1.00 |
| flipflop | The probability of trial flip-flopping | 0.000 | 0.000 | 0.000 | 0.001 | 0.000 | 1.000 |

*Note: Q1: 1^st^ quartile; Q3: 3^rd^ quartile.*

1. Results of simulations under the ‘larger difference’ scenario.

Supplementary Table 2. A summary of the output from 5,000 simulated trial datasets under the ‘larger difference’ scenario.

| Variable | Description of the variable | Minimum | Q1 | Median | Mean | Q3 | Maximum |
| --- | --- | --- | --- | --- | --- | --- | --- |
| nevents0 | Number of events in the control group | 14 | 18 | 26 | 25 | 30 | 49 |
| nevents1 | Number of events in the treatment group | 1 | 3 | 8 | 8 | 10 | 20 |
| pevents0 | Proportion of events in the control group | 0.05 | 0.09 | 0.10 | 0.10 | 0.11 | 0.26 |
| pevents1 | Proportion of events in the treatment group | 0.00 | 0.02 | 0.03 | 0.03 | 0.04 | 0.07 |
| sample_size | Sample size | 129 | 393 | 584 | 507 | 584 | 584 |
| interim_time | Time at the interim (days) | 123 | 259 | 300 | 306 | 349 | 570 |
| interim_or | Odds ratio at the interim | 0.04 | 0.16 | 0.29 | 0.30 | 0.40 | 1.26 |
| interim_lci | Lower CI for OR at interim | 0.00 | 0.04 | 0.09 | 0.10 | 0.14 | 0.51 |
| interim_uci | Upper CI for OR at interim | 0.21 | 0.50 | 0.79 | 0.81 | 1.04 | 3.22 |
| interim_p | P-value for any difference between treatments at the interim analysis | 0.00 | 0.00 | 0.02 | 0.06 | 0.07 | 1.00 |
| interim_stop | Whether the trial would have stopped at the interim (based on decision criteria at interim) | 0.00 | 0.00 | 0.00 | 0.28 | 1.00 | 1.00 |
| final_or | Odds ratio at final analysis | 0.03 | 0.21 | 0.28 | 0.29 | 0.36 | 1.06 |
| final_lci | Lower CI for odds ratio | 0.00 | 0.08 | 0.12 | 0.13 | 0.16 | 0.54 |
| final_uci | Upper CI for odds ratio | 0.13 | 0.45 | 0.58 | 0.61 | 0.72 | 2.08 |
| final_p | P-value for treatment difference at final analysis | 0.00 | 0.00 | 0.00 | 0.01 | 0.01 | 1.00 |
| final_stop | Whether the trial is conclusive at final analysis | 0.00 | 1.00 | 1.00 | 0.93 | 1.00 | 1.00 |
| stop | Whether the trial was conclusive (at the interim or at the final analysis) | 0.00 | 1.00 | 1.00 | 0.93 | 1.00 | 1.00 |
| flipflop | The probability of trial flip-flopping | 0.00 | 0.00 | 0.00 | 0.00 | 0.00 | 1.00 |

*Note: Q1: 1^st^ quartile; Q3: 3^rd^ quartile.*

1. Example code to obtain operating characteristics

In this appendix, we provide the example code to obtain the operating characteristics from the results of the trial simulations.

simno <- 5000 *#Number of simulations/trials*

*# Null hypothesis, note event probability is the same under null and alternative hypotheses.*

p_null <- c(p0,p0)

results_null <-runMultipleTrialsParallel(simno, seed, n, recruit_period, p_null, events_at_interim, alpha_interim, alpha_final)

*#Type I error rate: Probability of trial success under the null scenario*

typeIerror<-results_null$results_summary[“Mean”,”stop”]

*######################################*

*# Alternative hypothesis (Expected treatment effect; p0=0.10, p1=0.04).*

results_alternative <-runMultipleTrialsParallel(simno, seed, n, recruit_period, p, events_at_interim, alpha_interim, alpha_final)

*#Power: Probability of trial success under the alternative*

powerTrial<-results_alternative$results_summary["Mean","stop"]

*#flip-flop probability*

flipFlop<-results_alternative$results_summary["Mean","flipflop"]

*#0.001*

*#Average sample size*

sampleSize<-results_alternative$results_summary["Mean","sample_size"]

*#545*

*#Interim stop*

InterimStop<-results_alternative$results_summary["Mean","interim_stop"]

*#0.13*

*#Estimated average treatment effects from final analysis*

FinalResults<-results_alternative$results_summary["Mean",c("final_or","final_lci","final_uci","final_p")]

*#final_or final_lci final_uci final_p*

*#0.39233355 0.18859475 0.77356003 0.04070523*

*#Results*

print(paste0("Type I error rate is ",round(typeIerror,2)))

print(paste0("Power is ", round(powerTrial,2)))

print(paste0("Flip-flop probability is ", round(flipFlop,3)))

print(paste0("Average sample size is ", round(sampleSize)))

print(paste0("Probability of stopping at interim is ", round(InterimStop,2)))

1. Stata code

In this appendix, we provide the Stata code for reproducing the simulations for the simplified PIMS trial that we have presented in the paper using R.

********************************************************************************

* pims_sim.do

*

* Simulation program for the simplified PIMS trial

********************************************************************************

cd "C:\Users\katherine.lee\ownCloud\CEBU1-Kate_Lee\Kaushala\Simulation tutorial\"

clear all

set more off

********** Building block 1: Simulate treatment assignments **********

* Inputs: n - trial maximum sample size

cap prog drop randomisation

prog define randomisation

args n

* Set up the observations

clear

set obs `n'

* Generate randomisations using block randomisation with block size of 4

egen block = seq(), block(4)

bysort block: egen arm = seq(), to(2)

gen trt=arm-1

drop arm

gen random = uniform()

sort block random

gen obs_no=_n

drop block random

sort obs_no

order obs_no trt

end

* To run command

randomisation 584

********** Building block 2: Simulate accrual times of participants from a uniform distribution **********

* Inputs: n - trial maximum sample size

* recruit_period - a numeric value, the length of the recruitment period in days

cap prog drop accrual

prog define accrual

args n recruit_period

* Set up the observations

clear

set obs `n'

* Generate recruitment times (recruiting 310/wk means that recruiting 8062 would take 182 days)

gen accrual_time=round(runiform()*`recruit_period' + 0.5)

sort accrual_time

gen obs_no=_n

sort obs_no

end

* To run command

accrual 584 928

********** Building block 3: Generate participant outcomes **********

* Inputs: p0 - proportion with the event in the control arm

* p1 - proportion with the event in the intervention arm

* Note, needs to be run after randomisation

cap prog drop trialdata

prog define trialdata

args p0 p1

*** generate outcomes from a binomial distribution

gen disease=rbinomial(1,`p0') if trt==0

replace disease=rbinomial(1,`p1') if trt==1

end

* To run command (after running randomisation)

randomisation 584

trialdata 0.10 0.04

********** Building block 4: identify the data available at the interim analysis - at 100 events **********

* Inputs: none

* Note, needs to be run after generating the trial data

cap prog drop interim

prog define interim, rclass

gen dis_count=sum(disease)

sum obs_no if dis_count==20

return scalar interim_time=r(min)

gen dis_int=disease if obs_no<=r(min)

end

* To run command (after running trialdata)

interim

********** Building block 5: analyse the trial data **********

* Inputs: none

* Note, needs to be run after generating the trial data and identifying data available at the interim

cap prog drop analysis

prog define analysis, rclass

syntax, type(string)

qui{

if "`type'"=="interim" {

local outcome="dis_int"

}

if "`type'"=="final" {

local outcome="disease"

}

logistic `outcome' trt

mat results=r(table)

return scalar `type'_or=results[1,1]

return scalar `type'_lci=results[5,1]

return scalar `type'_uci=results[6,1]

return scalar `type'_p=results[4,1]

if "`type'"=="interim" {

if results[4,1]<0.005 {

return scalar interim_stop=1

}

else {

return scalar interim_stop=0

}

}

if "`type'"=="final" {

if results[4,1]<0.045 {

return scalar final_stop=1

}

else {

return scalar final_stop=0

}

}

}

end

* To run command (note )

analysis, type(interim)

analysis, type(final)

********** Running the simulations **********

* Inputs: seed - set to ensure reproducability

* simno - number of simulations

* n - trial maximum sample size

* recruit_period - a numeric value, the length of the recruitment period in days

* p0 - proportion with the event in the control arm

* p1 - proportion with the event in the intervention arm

cap prog drop simulations

prog define simulations

args seed simno n recruit_period p0 p1

clear

* set seed to ensure reproducibility

set seed `seed'

*** Set up postfile for results

cap frame drop sim_res

frame create sim_res simno pevents0 pevents1 interim_time interim_p interim_stop ///

final_or final_lci final_uci final_p final_stop

*local nrep = `simno'

local displaydots = 5

* Loop over the required number of simuations

forvalues i=1(1)`simno' {

clear

local sim_no=`i'

* Display the number of simulations conducted as dots on the screen

if mod(`i', `displaydots')==0 {

noisily disp _continue `i' " " // _continue - keep displaying on the same line

}

qui{

* Randomise participants

randomisation `n'

tempfile dataset

save `dataset', replace

* Generate accrual

accrual `n' `recruit_period'

* Merge randomisation and accrual data

merge obs_no using `dataset'

assert _merge==3

drop _merge

save `dataset', replace

* Generate trial outcomes

trialdata `p0' `p1'

save `dataset', replace

* Indetify timing of interim analysis

interim

local interim_time=r(interim_time)

* May wishe to capture proportion with event at study completion

forvalues i=0(1)1 {

preserve

keep if trt==`i'

count if disease==1

local pevents`i'=r(N)/_N

restore

}

* Conduct the interim and final analysis

analysis, type(interim)

local interim_p=r(interim_p)

local interim_stop=r(interim_stop)

analysis, type(final)

* Post results

frame post sim_res (`sim_no') (`pevents0') (`pevents1') ///

(`interim_time') (`interim_p') (`interim_stop') ///

(r(final_or)) (r(final_lci)) (r(final_uci)) (r(final_p)) (r(final_stop))

}

}

frame change sim_res

frame sim_res: save sim_res.dta, replace

end

* To run command - as powered scenario (p0 = 0.04, p1 = 0.10), 10000 simulations

simulations 64747545 5000 584 928 0.10 0.04

/*

*** Summary of results ***

Variable | Obs Mean Std. dev. Min Max

-------------+---------------------------------------------------------

simno | 5,000 2500.5 1443.52 1 5000

pevents0 | 5,000 .0998377 .0175414 .0445205 .1780822

pevents1 | 5,000 .0401322 .0116266 .010274 .0856164

interim_time | 5,000 285.0512 60.88472 117 520

interim_p | 4,998 .1437611 .2119089 .0013993 1

interim_stop | 5,000 .1244 .3300703 0 1

final_or | 5,000 .3918078 .1449251 .0843426 1.286724

final_lci | 5,000 .1949015 .080866 .0255247 .6887212

final_uci | 5,000 .7917602 .2611983 .244668 2.538928

final_p | 5,000 .0419466 .1021775 7.00e-07 1

final_stop | 5,000 .8072 .3945368 0 1
